# Supplementary material for: Immobilization of Enological Pectinase on Magnetic Sensitive Polyamide Microparticles for Wine Clarification
Source: Foods. 2024 Jan 28;13(3):420. doi: 10.3390/foods13030420 (PMC10855548; doi:10.3390/foods13030420)
Supplement: Supplementary file 1 [file foods-13-00420-s001.zip › foods-2811704-supplementary.pdf]

Immobilization of Enological Pectinase on Magnetic Sensitive Polyamide Microparticles for Wine Clarification

Sandra Cristina Oliveira, Nadya Vasileva Dencheva * and Zlatan Zlatev Denchev

IPC—Institute for Polymers and Composites, Campus of Azurém, University of Minho, 4800-058 Guimarães, Portugal; b8223@dep.uminho.pt (S.C.O.); denchev@dep.uminho.pt (Z.Z.D.)

* Correspondence: nadiad@dep.uminho.pt

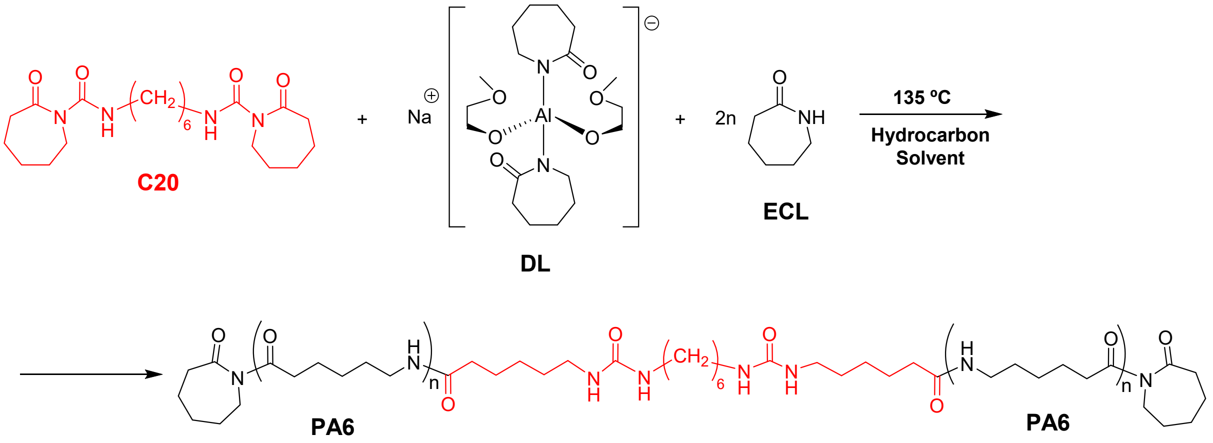

Figure S1. Chemical reactions occurring during AAROP of ε-caprolactam (ECL) to neat PA6 MP. The active substance of the AAROP activator is designated as C20; The chemical structure of the AAROP initiator dicaprolactamato-bis-(2-methoxyethoxo)-aluminate (DL) is presented wherein R = $OCH_2CH_2OCH_3$.

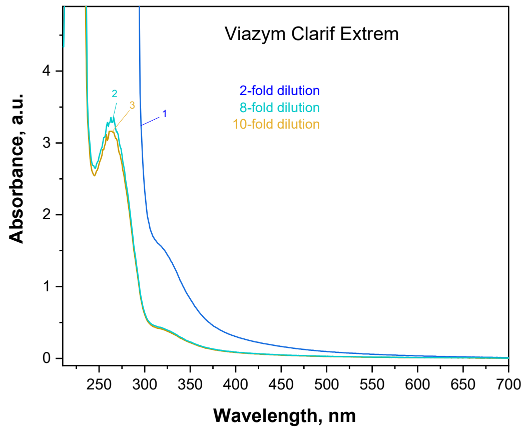

Figure S2. Determination of the optimal dilution of the enzymatic preparation that enables direct UV/VIS quantifion of the peak at 263 nm.

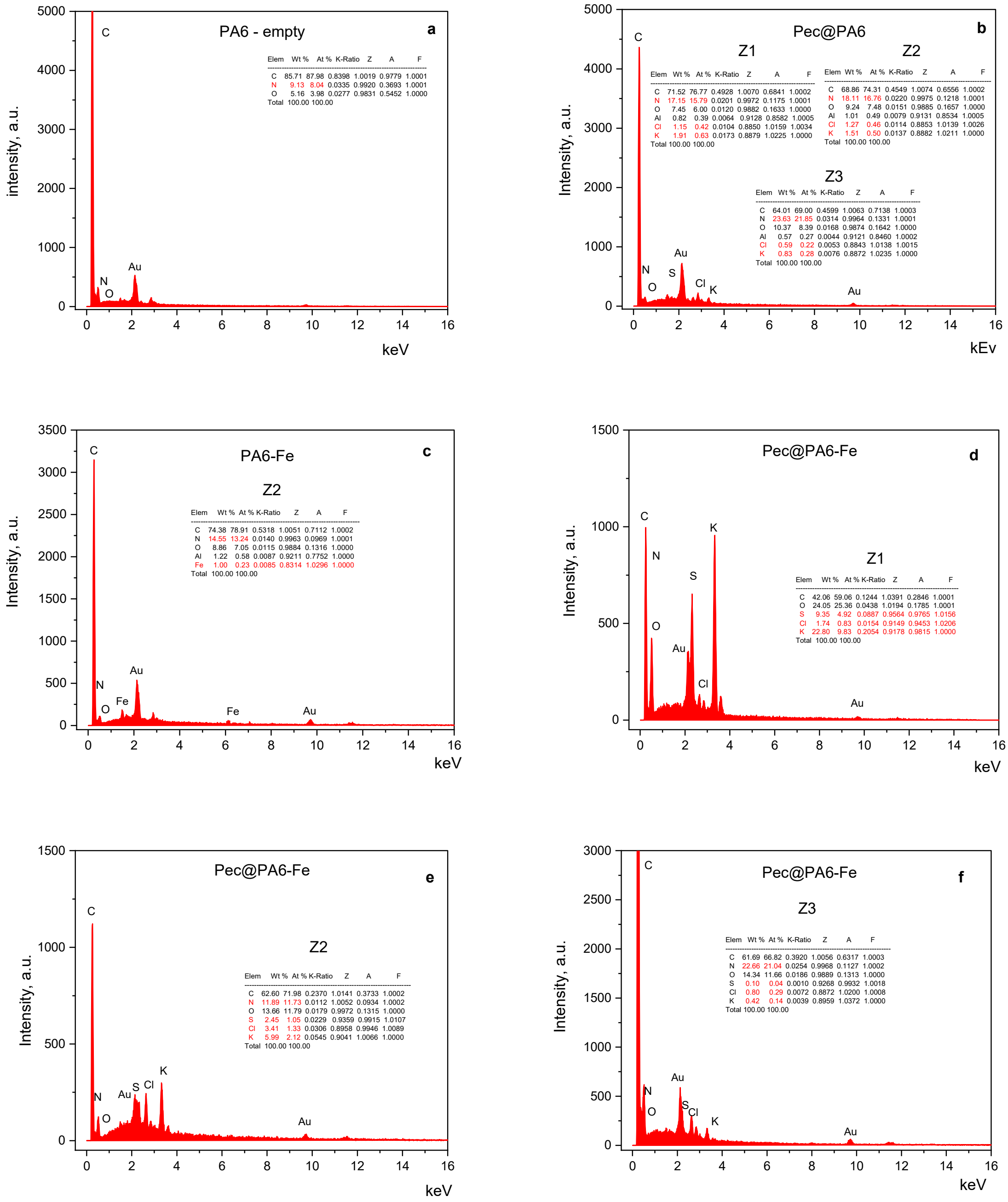

Figure S3. EDX results about the composition of empty supports and PEC@PA MP complexes. PA6 = empty PA6 support particles; PA6-Fe = PA6-Fe empty support particles; Pec@PA6 = Viazym-immobilized PA6 MP; Pec@PA6-Fe = Viazym-immobilized PA6-Fe MP. The specific sites Z_i where the EDX was performed are indicated in the main manuscript, Figure 1.

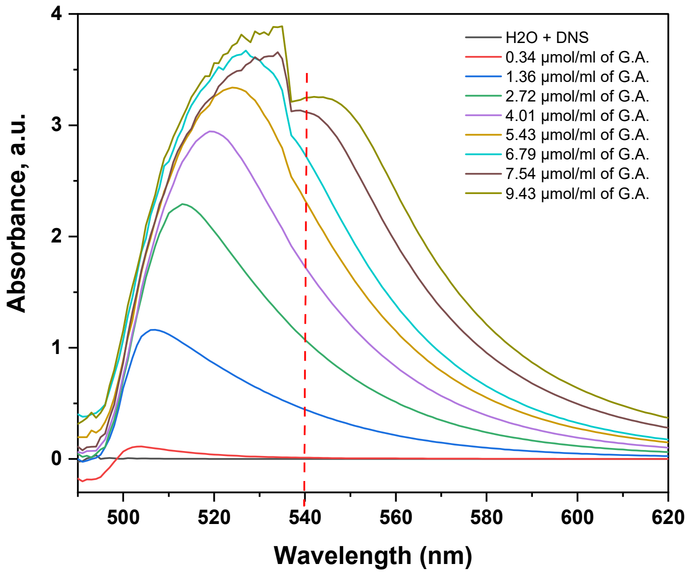

Figure S4. Evolution of the UV/VIS absorption maxima of various concentrations of galacturonic acid treated with the DNS reagent. G.A. = galacturonic acid. The vertical line at 540 nm indicates the position of the peak of the resulting 5-amino-3-nitrosalycilic acid colored product used in the construction of the standard calibration plot used in the pectinase activity assay.

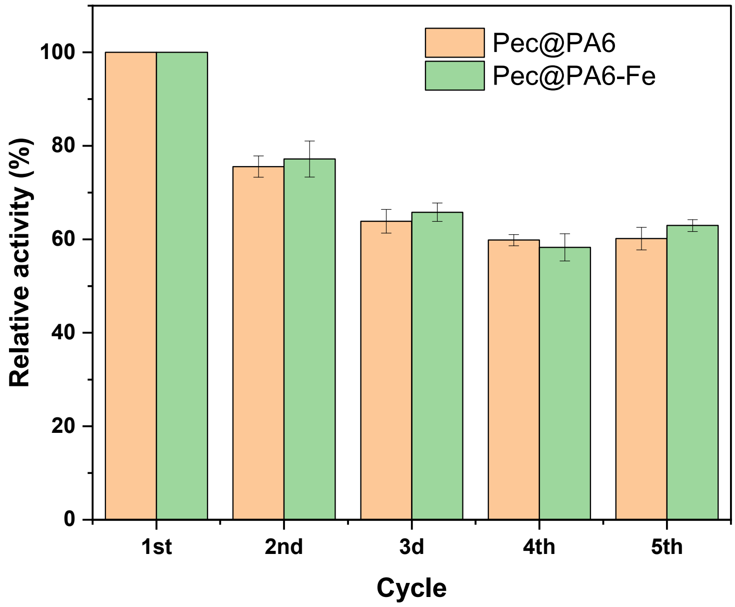

Figure S5. Relative activity of Pec@PA6 and Pec@PA6-Fe during 5 consecutive cycles
